# Using Patient Blogs on Social Media to Assess the Content Validity of Patient-Reported Outcome Measures: Qualitative Analysis of Patient-Written Blogs

**DOI:** 10.2196/43210

**Published:** 2023-07-28

**Authors:** Diana M J Delnoij, Meggie Derks, Laura Koolen, Shuka Shekary, Jozua Suitela

**Affiliations:** 1 Erasmus School of Health Policy & Management Erasmus University Rotterdam Rotterdam Netherlands; 2 National Health Care Institute (Zorginstituut Nederland) Diemen Netherlands

**Keywords:** patient stories, patient-reported outcome measure, PROM, social media, patient stories, narrative, patient story, storytelling, blogger, experiential, experience, content validity, content analysis, qualitative, cross sectional, cross-sectional, chronic disease, noncommunicable diseases, NCD, rheumatoid arthritis, Parkinson disease, diabetes mellitus, diabetes, type II diabetes, cancer, breast cancer, oncology, International Consortium for Health Outcome Measurement, ICHOM, data dictionary, Health Assessment Questionnaire, HAQ, Parkinson Disease Quality of Life Questionnaire, PDQ, inductive, inductive code

## Abstract

**Background:**

Patient-reported outcome measures (PROMs) are questionnaires that measure patient outcomes related to quality of life, health, and functioning, and are increasingly used to assess important outcomes from the patient’s perspective. For PROMs to contribute to better health and better care, it is vital that their content validity be adequate. This requires patient involvement in various steps of PROM development. PROM developers not only recognize the benefits of patient involvement but also report difficulties in recruiting patients and experience patient involvement as time-consuming, logistically challenging, and expensive.

**Objective:**

This study seeks to explore different strategies for disclosing the experiential knowledge of patients, namely through analyzing patient stories on the web and social media. The research questions are as follows: (1) how do bloggers living with a disease experience their health-related quality of life? (2) How are these experiences reflected in the domains and items of PROMs related to their disease?

**Methods:**

First, a qualitative analysis of blogs written by patients was performed. Second, subthemes and underlying codes resulting from this qualitative analysis were systematically compared with the domains and items in PROMs for the respective diseases that the bloggers write about. Blogs were identified via the Google search engine between December 2019 and May 2021.

**Results:**

Bloggers describe a wide range of experiences regarding their physical functioning and health; mental well-being; social network and support; daily life, education, work, and leisure; coping; and self-management. Bloggers also write about their positive and negative experiences with health care delivery, the organization of health care, and health care professionals. In general, patients’ experiences as described in blogs were reflected in the domains and items of the PROMs related to their disease. However, except for diabetes mellitus, in all the sets of PROMs, potentially missing topics could be identified. Similarly, with the exception of Parkinson disease, all PROMs address issues that patients did not write about in their blogs and that might therefore be redundant.

**Conclusions:**

Web-based patient stories in the form of blogs reveal how people living with a certain disease experience their health-related quality of life. These stories enable analyses of patients’ experiences that can be used to assess the content validity of PROMs. This can be a useful step for researchers who are looking for sets of measuring instruments that match their purposes.

## Introduction

Value-based health care (VBHC) has globally been embraced as a concept that guides innovation and improvement in health care delivery [1-4]. In VBHC, value is defined as “the health outcomes achieved per dollar spent” [5]. Therefore, defining outcomes that matter to patients is essential [6]. To that end, the International Consortium for Health Outcomes Measurement (ICHOM) has developed standard sets of outcomes for different conditions in order to support VBHC [7]. ICHOM sets usually combine clinical measures with patient-reported outcome measures (PROMs).

PROMs are questionnaires that measure patient outcomes related to quality of life, health, and functioning, and are increasingly used to assess important outcomes from the patient’s perspective [8]. PROMs can either be generic or disease-specific. Generic PROMs can be used to measure health and health-related quality of life for various conditions, whereas disease-specific PROMs aim to measure how a patient experiences the consequences and severity of a specific condition [9]. Through measuring health outcomes and health-related quality of life from the perspective of patients, PROMs tap into the experiential knowledge that patients possess. This experiential knowledge on health and health care is increasingly seen as a source of information that is relevant for improving the quality of care [10].

For PROMs to contribute to better health and better care, it is vital that their content validity be adequate and that, in other words, they truly measure outcomes that matter to patients. To assure the content validity of PROMs, the content of PROMs needs to be examined by the targeted patient group [11,12]. This requires patient involvement in various steps of PROM development: from defining which outcomes should be measured, as well as deciding on the items that will construct those outcomes and testing the formulation of those items [13]. However, Wiering et al [10] showed that 1 of 4 PROMs has been developed without patient involvement, whereas patient involvement in deciding which outcomes to measure was limited to 1 in 10 PROMs. Patient involvement is most common in item development and often takes the form of focus groups or interviews. PROM developers recognize the benefits of patient involvement. However, they also report difficulties in recruiting a diverse group of patients and experience patient involvement as time-consuming, logistically challenging, and expensive [14].

Therefore, it is interesting to explore different interpretivist strategies for disclosing the experiential knowledge of patients, namely through analyzing patient stories on the web and social media. van de Bovenkamp et al [15] argue that patient stories can enable professionals to gain a better understanding of their patients, enhance the strength of patient organizations, and can contribute to improving quality of care. Apart from that, patient stories provide insight into the complex, layered view that patients have of their disease and of the way it influences their lives [15]. Furthermore, patient stories on social media are a readily available source of information. Several studies have explored the potential of social media as a data source to assess patient-important outcomes. For example, Rothman et al [16] argue that using social media as a source is less time-consuming, less expensive, and has the ability to reach more patients or caregivers compared with traditional methods of patient involvement.

McCarrier et al [17] showed that web-based social networks can be used to determine which outcomes are important to patients with leukemia. Similarly, Kalf et al [18] showed that the analyses of web-based melanoma patient forums can reveal relevant aspects of health-related quality of life and could be used not only to improve the content validity of PROMs but also to prioritize topic selection in the scoping phase of the value assessment of health technologies.

Building on these suggestions, therefore, this study aims to explore the use of patient stories in the form of blogs published on the web for assessing the content validity of PROMs. The research questions are as follows: (1) how do bloggers living with a disease experience their health-related quality of life? (2) How are these experiences reflected in the domains and items of PROMs related to their disease?

## Methods

### Qualitative Approach

The research questions are addressed first through a qualitative analysis of blogs written by patients. Second, subthemes and underlying codes resulting from this qualitative analysis are systematically compared with the domains and items in PROMs for the respective diseases that the bloggers write about.

### Sampling Strategy

To demonstrate the feasibility of using blogs to assess the content validity of PROMs, blogs on 3 different chronic disease are used, namely rheumatoid arthritis, Parkinson disease, and diabetes mellitus. In addition, blogs of patients with breast cancer and its survivors have been analyzed. Blogs were identified between December 2019 and May 2021 in separate research projects coordinated by the first author (DMJD). The individual studies took place as master thesis projects of the master programs Health Economics, Policy and Law and Health Care Management of the Erasmus School of Health Policy & Management. The choice of the diseases was made by the individual students, who were free to pick a topic of interest provided that an ICHOM standard set of outcome measures was available for the chosen disease category.

ICHOM standard sets include recommendations about which PROMs to use. Data on recommended PROMs were derived from ICHOM Connect [19-23], specifically the reference guides and data dictionaries provided for the following standard sets: breast cancer, inflammatory arthritis, Parkinson disease, and diabetes. The reference guides and data dictionaries are available free of charge; however, registration is required to access them. In the reference guides, links are provided to the PROM questionnaires.

The analyses for breast cancer and diabetes mellitus included all the PROMs that are recommended by ICHOM. However, the list of PROMs that ICHOM recommends for inflammatory arthritis is very long. Therefore, for practical reasons, we choose to focus on the 2 PROMs from that list, which are most frequently used by Dutch physicians to measure outcomes of care in patients with rheumatoid arthritis, namely the Health Assessment Questionnaire and the Short-Form 36 (SF-36) [23]. Alternatively, ICHOM recommends only 1 PROM for Parkinson disease, namely the Parkinson Disease Quality of Life Questionnaire 8 (PDQ-8) [24]. This is a shortened version of the PDQ-39 developed by Peto et al [25]. According to Marinus et al [26], it is the most widely used PROM in care for patients living with Parkinson disease. For that reason, the PDQ-39 has also been included in the analysis.

### Data Collection Methods

The data on breast cancer blogs were collected and coded by MD and DMJD, rheumatoid arthritis by LK and DMJD, Parkinson disease by JS and DMJD, and diabetes mellitus by SS and DMJD. Blogs were identified using Google’s search engine, and bloggers’ contributions were entered into the analysis in batches. For example, the analysis of bloggers with breast cancer started with the 5 most recent blog posts of 20 different bloggers. This process continued until data saturation was reached (see analysis).

All projects included blogs written by Dutch patients in order to be able to draw conclusions on the validity of the respective PROMs in the context of the Dutch health care system. We used blogs that were available in the public domain (see the Ethical Consideration section). Furthermore, for patients living with rheumatoid arthritis, data collected from blogs were complemented with patient stories based on interviews, to account for potential bias caused by the fact that patients with severe rheumatoid arthritis cannot write blogs anymore [27].

See Multimedia Appendix 1 for more details on the data collection, inclusion and exclusion criteria, search strings, number of blogs coded, and PROMs included in the comparison.

### Data Processing and Analysis

Full texts of blogs were imported into the ATLAS.ti software (ATLAS.ti Scientific Software Development GmbH) using identification numbers or names of bloggers, depending on whether a blogger chose to be quoted as an author (see the Ethical Consideration section). In ATLAS.ti, unstructured, raw data from blogs were analyzed using open inductive coding in an iterative process. This analysis resulted in the identification of themes and subthemes that have been used to explore how bloggers experience their health-related quality of life. Health-related quality of life is a broad concept relating to the effect of a particular health status on a person’s well-being [28]. This includes both the physical ability and the mental ability to function properly, and it can be measured via PROMs [29]. In this study, health-related quality of life is defined from the bloggers’ subjective perception of the impact of disease and of its treatment on physical, psychological, and social aspects of daily life. Although aspects related to health care experiences conventionally do not belong in the definition of health-related quality of life, previous research showed that for patients, quality of life and experiences with health care are entwined [30]. For that reason, these aspects have been included.

As the initial coding process took place in separate research projects and data were inductively coded from the perspective of the bloggers, separate codebooks exist for the 4 disease categories. For the purpose of summarizing the findings in this paper (Table 1), disease-specific codes have been aggregated into 6 global categories: physical functioning and health; mental well-being; social network and support; daily life, education, work, and leisure; coping and self-management; and experiences with health care. The codebooks per disease category are presented in Multimedia Appendix 2. Multimedia Appendix 3 specifies which codes have been assigned to which category of Table 1.

**Table 1:** Percentage of quotations per category per patient group.^a^

Category	Rheumatoid arthritis	Parkinson disease	Breast cancer	Diabetes mellitus
Physical functioning and health	58	52	25	25
Mental well-being	23	78	28	23
Social network and support	12	28	8	20
Daily life, education, work, and leisure	46	50	7	19
Coping and self-management	2	46	12	34
Experiences with health care	38	20	27	39
Other	1	—^b^	5	4
Total number of quotations	1148	787	1549	497

^a^Many text fragments contain >2 so the percentage adds up to >100%.

^b^Not available.

As a consequence of the nature of the primary research projects, which were relatively small master thesis studies, the check of the supervising author (DMJD) on the coding conducted by each of the other authors (MD, LK, SS, and JS) focused on (1) obtaining consensus about the inclusion of text fragments based on the broad and subjective definition of health-related quality of life, (2) reaching consensus about the way in which codes have been grouped into subthemes and themes, and (3) consistent application of the codes within each of the separate data sets. The unique codes themselves have not been applied by 2 of the authors separately in order to be able to calculate the level of agreement. Furthermore, it is important to note that the coding took place before and separate from the comparison with the various PROMs because we wanted to interpret themes and subthemes in the qualitative data without being influenced by domains and items in the PROMs. In all the research projects, data collection and analysis continued until no new topics were extracted from the data, implying that data saturation had occurred.

The comparison with the PROMs was guided by the criteria for content validity described by Terwee et al [12]. Specifically, we focused on two criteria: (1) Are the included items relevant for the target population of interest? (2) Are no key concepts missing? The comparison was done on the level of themes (qualitative data) and domains (PROMs), as well as on the level of subthemes or codes (qualitative data) and items (PROMs).

### Ethical Consideration

In the Erasmus School of Health Policy & Management, research conducted by students for their master thesis is exempt from review by the internal ethical committee. The ethical assessment is the responsibility of the supervisor. We handled this as follows: all the blogs that have been used were published in the public domain on the web platform. The first research project focused on bloggers with Parkinson disease and took place in 2020. In that specific case, we argued that blogs were made public by their authors and therefore could be used for the purpose of academic quality of life research. However, in subsequent projects that took place in 2021 on rheumatoid arthritis, diabetes mellitus, and breast cancer, discussions within our research team led us to take a different viewpoint. Following the line of reasoning described by Snee [31], it is possible to view bloggers posting blogs in the public domain as either human participants—in which case, informed consent for using their blogs should be obtained—or as authors—in which case, their blogs should be adequately referenced. In the 3 research projects on rheumatoid arthritis, diabetes mellitus, and breast cancer, we decided to leave the decision whether they should be seen as participants or authors essentially up to bloggers themselves. Therefore, all the platforms and bloggers whose blogs we intended to include were contacted asking them (1) whether we could use their blog posts and (2) whether they wanted us to use potential quotes with referencing, so as to acknowledge their rights as authors. Blogs of platforms or authors who did not grant permission for use were of course excluded. However, blogs of authors who did not reply to our mail have been included in the analyses but without using quotes from their blogs in the *Results* section.

## Results

### Qualitative Analysis

Table 1 summarizes how bloggers experience their health-related quality of life. This table shows that bloggers describe a wide range of experiences regarding their health and physical functioning; their mental well-being; their social network and support; how they experience their illness in daily life, education, work, and leisure; and how they cope and self-manage. Experiences with health care conventionally do not fall under health-related quality of life. However, for reasons explained earlier, quotations in which bloggers describe their experiences with health care delivery, the organization of health care, and health care professionals have been coded in all of the projects.

Although bloggers from all 4 patient groups cover this broad range of topics, their specific experiences differ. Bloggers writing about rheumatoid arthritis frequently discuss topics concerning limitations in physical functioning, for example, pain, that cause inconveniences in daily life. Some patients with rheumatoid arthritis are not able to walk for a long time or to lift heavy objects; in other patients, the limitations manifest themselves in smaller everyday activities: “For example, because of my limited hand function, I am a little nervous with every new toilet outside the home about whether I can open the locked toilet door again. Yes, I sometimes fail” [32]. Despite their rheumatoid arthritis, bloggers write about still being engaged in various activities, such as work, household and grocery shopping, and in leisure activities. Even though several bloggers with rheumatoid arthritis reported to be partially or completely incapacitated for paid work, they often still perform voluntary work.

Bloggers with Parkinson disease often write about how they feel. Although they do mention positive attitudes, like staying hopeful and enjoying life, they also say to be struggling with feelings of sadness, depression, anxiety, mood changes, and negative thoughts. Living with the disease provokes emotions such as feeling insecure, vulnerable, and ashamed. With respect to physical problems, bloggers with Parkinson disease write, for example, about “fatigue.” One blogger describes how she struggles:

I always have a low energy level. On a good day I have about 50 per cent of the energy of a woman that isn’t sick. So that isn’t much. I get tired easily and after an intensive day I need a whole day to recover and I won’t be able to work at all.

Parkinson disease blogger 7, anonymous

Other health issues described include symptoms like rigidity, postural instability, bradykinesia, tremor, pain, dyskinesia, the so-called freezing, loss of balance, and hypomimia.

For patients with breast cancer and its survivors, feelings are an important issue too. For example, bloggers mention feelings of sadness; anxiety or fear; depressive feelings; and feelings of frustration, anger, loneliness, shame, guilt, and insecurity. Several bloggers write about trust in their own body and about fear for relapses: “I am terrified that it will return” [33]. Concerning their physical health, bloggers with breast cancer write about the side effects of treatment. Patients with breast cancer experience different side effects from different treatments, for example, damaged skin and fluid in the breast (for radiation therapy); osteoporosis and the growth of fuzzy hair on the body (for hormone therapy); and the whole range of chemotherapy side effects such as hair loss, nausea, hot flushes, weight gain, stool problems, and pain in arteries or bones. Also, having “a chemo brain” is frequently mentioned as a side effect, having a haze in one’s head, or forgetting things. Besides side effects from treatment, bloggers describe other physical problems, such as having a weak immune system, lack of fitness, or exhaustion.

Bloggers writing about diabetes mellitus describe how their condition forms an integral part of daily life. Diabetes is “a continuous presence, a presence that is just as normal as eating, drinking, and sleeping” (diabetes mellitus blogger 11, anonymous). Self-management and controlling blood sugar levels never stop. Patients have to take responsibility all the time, for example, to make sure to be prepared for a hypo- or hyperglycemia.

When I go for a walk, I always make sure I have some important items with me. It seems terrible to me to get a hypo while being on the way and not having anything to eat or drink with me.

Diabetes mellitus blogger 7, anonymous

### Comparison With PROMs

Table 2 shows the comparison of domains and subthemes that have been identified in the blogs with the domains and items in the PROMs for the specific diseases. There are 3 possibilities: domains and items in the PROM match with domains and subthemes of the blogs, domains and items in the PROM do not match with domains and subthemes of the blogs (so they do occur in the PROM but not in the blogs), and domains and items are missing in the PROM (so they are mentioned in the blogs but are not covered in the PROM).

The results of the comparison for breast cancer show that the majority of topics addressed in the BREAST-Q and in the European Organization for Research and Treatment of Cancer Core Quality of Life Questionnaire (EORTC QLQ-C30) are also mentioned in the blogs. Only a minority of items in those 2 PROMs are not matched by issues discussed in the blogs. However, 18 of the items that the EORTC Quality of Life Questionnaire—Breast Cancer (EORTC QLQ-BR45) addresses are not described by bloggers with breast cancer. Vice versa, coping strategies and problems with fertility and pregnancy support are mentioned by bloggers but are not covered by any of the 3 breast cancer PROMs that are recommended by ICHOM.

For rheumatoid arthritis, the comparison of the Health Assessment Questionnaire and SF-36 shows that many of the topics addressed in those PROMs were also coded in the qualitative analyses of the blogs. There is one exception: both PROMs address personal care; however, this issue is not mentioned in the blogs. Alternatively, there are 3 topics that are discussed in blogs but are missing in the PROMs, namely side effects of drugs, flares, and experiences with treatment.

All the items covered in the PDQ-39 and PDQ-8 for Parkinson disease are also mentioned in the blogs written by patients with Parkinson disease. However, bloggers writing about Parkinson disease discuss a whole range of issues that are missing in the PROMs, namely fulfilment, impact on social life, perceived quality of care, personality change, relationship, self-management, and sleep disorder.

Finally, the 5-item World Health Organization Well-Being Index, the 9-item Patient Health Questionnaire, and the Problem Areas in Diabetes together cover all the issues that are discussed by bloggers writing about diabetes mellitus. There are no missing domains. However, all 3 of the diabetes PROMs contain questions about topics that bloggers have not mentioned, for example, whether daily life is filled with interesting things (5-item World Health Organization Well-Being Index); having little interest or pleasure in doing things, trouble concentrating on things, and moving or speaking slowly, or being fidgety or restless (9-item Patient Health Questionnaire); and not having clear and concrete goals for diabetes care, feeling discouraged with diabetes treatment plan, feelings of deprivation regarding food and meals, or not knowing if mood or feelings are related to diabetes (Problem Areas in Diabetes).

**Table 2:** Content validity of PROMs^a^: comparison of domains and subthemes in blogs with domains and items in PROMs.

Domain		Breast cancer	Rheumatoid arthritis	Parkinson disease	Diabetes mellitus
**PROMs**		BREAST-Q	HAQ^b^	PDQ-39/PDQ-8^c^	WHO-5^d^
	Domains matching with subthemes blogs	Psychosocial well-being Sexual well-being Satisfaction with breast Satisfaction with nipple reconstruction Satisfaction with implants Breast animation deformity (implants) Physical well-being: chest Physical well-being: back and shoulder Adverse effects of radiation Satisfaction with information Satisfaction with information—breast surgeon Satisfaction with information—radiation oncologist Satisfaction with surgeon Satisfaction with office staff Satisfaction with medical team	Daily activities Physical health Pain Mobility	Activities of daily living Bodily discomfort Cognition Communication Emotional well-being Autonomy Social support Stigma	Feeling cheerful and in good spirits Feeling calm and relaxed Feeling active and vigorous Waking up fresh and rested
	Domains not matching with subthemes blogs	Satisfaction with back Physical well-being: abdomen Satisfaction with abdomen	Personal care	—^e^	Daily life filled with interesting things
**PROMS**		EORTC QLQ-BR45^f^	SF-36^g^	—	PHQ-9^h^
	Domains^i^ matching with subthemes blogs	Hair loss Upset by hair loss Ill or unwell feeling Hot flushes Headaches Feeling physically less attractive Feeling less feminine Dissatisfaction with body Worries about future health Pain in arm or shoulder Swollen breast Skin problems breast Pain in hands or feet Tingling in fingers or toes Problems with joints Stiffness in joints Pain in joints Aches or pain in bones Weight gain Problem of weight gain Satisfaction with cosmetic result of surgery Satisfaction with appearance of the skin of affected breast	Daily activities Physical health Psychological well-being Social activities Pain Fatigue Mobility General health		Feeling down, depressed, or hopeless Trouble falling or staying asleep, or sleeping too much Feeling tired or having little energy Poor appetite or overeating Feeling bad about oneself, or that one is a failure or let oneself or one’s family down Thoughts that one would be better off dead, or of hurting oneself
	Domains^i^ not matching with subthemes blogs	Dry mouth Different taste Painful, irritated, or watery eyes Problems looking at yourself naked Interest in sex Sexually active Sex is enjoyable Swollen arm or hand Problems raising or moving arm Excessive sweat Dizzy Soreness in mouth Reddening in mouth Aches or pain in muscles Dry vagina Discomfort in vagina Pain in vagina during sexual activity Dry vagina during sexual activity	Personal care		Little interest or pleasure in doing things Trouble concentrating on things Moving or speaking slowly, or being fidgety or restless
**PROMs**		EORTC QLQ-C30^j^	—	—	PAID^k^
	Domains^i^ matching with subthemes blogs	Trouble with strenuous activities Trouble taking a long walk Trouble taking a short walk The need to stay in bed during the day Limited in doing work or other activities Limited in pursuing hobbies or leisure time activities Short of breath Feeling pain Need to rest Feeling weak Lack of appetite Feeling nauseated Having vomited Being constipated Having diarrhea Feeling tired Pain interfered with daily activities Difficulty concentrating Feeling tense Feeling irritable Feeling depressed Having difficulties remembering things Condition or treatment has interfered with family life Condition or treatment has interfered with social activities Condition or treatment caused financial difficulties			Feeling scared about living with diabetes Uncomfortable social situations related diabetes care Feeling depressed about living with diabetes Feeling overwhelmed by diabetes Worrying about low blood glucose reactions Feeling angry about living with Diabetes Feeling constantly concerned about food and eating Worrying about the future and the possibility of serious complications Feelings of guilt or anxiety about getting off track with diabetes management Not “accepting” diabetes Feeling unsatisfied with diabetes physician Feeling that diabetes is taking up too much mental and physical energy every day Feeling that friends and family are not supportive of diabetes management efforts Coping with complications of diabetes Feeling “burned-out” by the constant effort needed to manage diabetes
	Domains^i^ not matching with subthemes blogs	Need help with eating, dressing, washing or using the toilet Trouble sleeping			Not having clear and concrete goals for diabetes care Feeling discouraged with diabetes treatment plan Feelings of deprivation regarding food and meals Not knowing if mood or feelings are related to diabetes
Domains missing in all PROMs		Coping strategies Fertility or pregnancy support	Side effect drugs Flares Experiences treatment	Fulfilment Impact on social life Perceived quality of care Personality change Relationship Self-management Sleep disorder	—

^a^PROM: patient-reported outcome measure.

^b^HAQ: Health Assessment Questionnaire.

^c^PDQ: Parkinson Disease Quality of Life Questionnaire.

^d^WHO-5: 5-item World Health Organization Well-Being Index.

^e^—: not available.

^f^EORTC QLQ-BR45: European Organization for Research and Treatment of Cancer Quality of Life Questionnaire—Breast Cancer.

^g^SF-36: Short-Form 36.

^h^PHQ-9: 9-item Patient Health Questionnaire.

^i^The EORTC QLQ-C30, EORTC QLQ-BR45, WHO-5, PHQ-9, and PAID consist of lists of items not grouped into domains; therefore, the analyses for these 2 PROMs took place at the level of items.

^j^EORTC QLQ-C30: EORTC Core Quality of Life Questionnaire.

^k^PAID: Problem Areas in Diabetes.

## Discussion

### Principal Results

This study aimed to explore the use of patient stories in the form of blogs to assess the content validity of PROMs. Our first research question was: How do bloggers living with a disease experience their health-related quality of life? The blogs cover a broad set of themes that reflect common domains of health-related quality of life [34] such as physical functioning, psychological well-being, social and role functioning, and health perceptions. In addition, bloggers write about their positive and negative experiences with health care delivery, the organization of health care, and health care professionals.

The second research question was: How are these experiences reflected in the domains and items of PROMs related to their disease? The analyses showed that, in general, patients’ experiences as described in blogs were reflected in the domains and items of the PROMs related to their disease. However, with the exception of PROMs for diabetes mellitus, for all the patient groups, potentially missing topics could be identified, that is, issues that were raised in blogs but are not addressed in PROMs. Similarly, with the exception of the PROMs for Parkinson disease, PROMs address issues that patients did not write about in their blogs. This is particularly the case for the ICHOM breast cancer and diabetes mellitus PROMs.

### Reflections and Comparison With Prior Work

The finding that bloggers with rheumatoid arthritis or Parkinson disease write about the limitations experienced in daily life might reflect the disabling, progressive course of rheumatoid arthritis and Parkinson disease and the effect this has on daily life [35-37]. Similarly, the findings of the qualitative analysis of the blogs are in line with previous research findings, for example, reported by Benkel et al [38]. They showed how patients with diabetes mellitus experience the impact on their daily life of having to check blood sugar levels every day, and how patients with rheumatoid arthritis experience the impact of pain on their daily life.

In the scientific literature, there are many reports of qualitative research aimed at understanding how patients experience their lives with chronic diseases [39-41]. According to Eatough and Shaw [42], this type of research can shed light on patients’ needs and contribute to health care practices that are tailored to the needs of individual persons. They argue that people are more than their symptoms and that qualitative research of people with Parkinson reveals that they do not only want treatment for their symptoms but also need practical support in daily living. Our study based on blogs has demonstrated that stories written by patients living with a disease can provide similar valuable insights. Reading such blogs can therefore be useful for health care practitioners or for medical and nursing students who wish to provide more person-centered care.

Our comparison with PROMs shows that it is probably more relevant to assess content validity at the level of an ICHOM set as a whole rather than for single PROMs in those sets. Content validity refers to the relevance of items for the construct of interest. If the construct of interest is outcomes that matter to patients, as is the case with the ICHOM, a set of PROMs need to be chosen that together will address all these outcomes. Even so, however, it remains questionable whether it is possible to capture the full range of patient experiences in a meaningful way. For example, in a discourse analysis, Steinmann et al [43] reported critical views on the standardization of health care and care outcomes in VBHC. One could argue that the reduction of experiences of patients into outcomes in (even a set of) PROMs might not do enough justice to their real experiences.

Nonetheless, this explorative study has shown the potential of using patient stories in the form of blogs for assessing the content validity of PROMs. Health-related quality of life themes could be identified in the blogs of all patient groups. This supports the use of generic PROMs such as the SF-36, the EuroQol 5D, or other global measures in ICHOM standard sets [44]. Similarities in the experiences of the 4 groups of patients are visible in issues such as fatigue, feeling tired, or even exhausted, or in negative feelings which are described, such as depression of fear. However, the qualitative analysis also revealed differences between patient groups that generic PROMs may fail to uncover. For example, experiencing limitations in physical function for a person with rheumatoid arthritis means dealing with pain, whereas for a person with diabetes, it involves always being prepared for a hypo- or hyperglycemia. Disease-specific PROMs might be better suited for capturing such subtleties.

Although some missing topics could be identified, overall, patients’ experiences as described in blogs are reflected in PROMs. However, the PROMs in the ICHOM breast cancer set and in the ICHOM diabetes mellitus set also address issues that were not identified in the blogs. This indicates that some of the questions are potentially redundant, depending perhaps on the stage of the patient journey in which the surveys are administered. The breast cancer PROMs, for example, contain questions that are relevant during treatment but maybe less relevant for patients who are long-term breast cancer survivors.

So, blogs provide a rich source of data as argued by van de Bovenkamp et al [15]. According to Murphy et al [45], this is not only the case for somatic diseases as the ones studied here but also for mental health issues. Although the analysis of blogs is time-consuming when done manually, text mining offers promising results and could potentially enable the processing of large volumes of web-based patient stories [46]. However, there are also downsides to the use of web-based patient stories. As described in the Methods section, there are ethical considerations to take into account. Using blogs in the most ethically responsible matter implies contacting all the authors and owners of websites. This is time-consuming and poses further ethical challenges in case an author does not respond.

Despite these challenges and the limitations described above, blogs have some advantages over using interviews or focus groups in the development of PROMs. Conducting interviews too is time-consuming, and interviews are a co-constructing method in which both interviewer and participant contribute to the conversation [47]. This implies that the interviewer can steer the conversation and the analysis [48]. Similarly, in focus groups, a form of group bias can occur, which requires a skilled and well-trained group facilitator, and participants may be reluctant to talk about sensitive topics [49].

All in all, in developing new PROMs from scratch, the use of more traditional methods such as interviews or focus groups is probably preferable because they offer better options to control the selection of participants and minimize bias. However, in many cases, researchers do not aim at developing a new PROM but at choosing the right PROMs from the large number of questionnaires that are available [50]. In this study, we demonstrated that an analysis of web-based patient stories can be useful in determining which set of measuring instruments reflect outcomes that matter to patients.

### Strengths and Limitations

The strength of our study lies in the systematic use of qualitative research to analyze the content validity of PROMs that included in ICHOM standard sets. Even though these standard sets aim to measure outcomes that matter, our analysis has shown that the sets may still miss topics that are potentially relevant for patients and address issues that might be redundant from the patient perspective.

A possible limitation of our study is that the initial coding of blogs took place in separate projects in which data were inductively coded from the perspective of the bloggers. This increases the internal validity of the codes per patient group, but it limits the possibilities of comparing codes across groups. However, comparison across patient groups was not the purpose of our study. Instead, we aimed to use the results of the qualitative analyses per patient group to assess the content validity of the PROMs that are used for that specific population.

In addition, it is difficult to verify the accuracy of the information that bloggers provide. It is possible that bias occurs because bloggers are more inclined to try to appeal to their readers rather than alienate them [51]. An inherent disadvantage of our approach is that we did not have much background information about the bloggers other than the information they provided in the blogs. This implies that information on clinically relevant details such as the stage of the disease is missing. In the case of bloggers with diabetes mellitus, for instance, it was not always clear whether a blogger had type 1 or type 2 diabetes. Even though treatment regimens differ for the 2 types of diabetes mellitus, bloggers with both type 1 and type 2, as well as bloggers for whom the type was unclear, were included in the analyses. The reason for doing so is that the ICHOM standard set from which the PROMs were derived is also intended to cover both diabetes mellitus type 1 and type 2.

Apart from that, the use of blogs also leaves a group of patients unheard of, for example, the group of older patients who are often not very comfortable with using the internet [52]. A blogger’s age is often unknown. However, several bloggers with breast cancer talked about fertility issues, indicating that probably the sample included comparatively younger patients with breast cancer.

### Conclusions

Web-based patient stories in the form of blogs reveal how people living with a certain disease experience their health-related quality of life. These blogs enable a rich description of patients’ experiences with their health and physical functioning, their feelings, side effects of treatment, and their coping with the disease in daily live. Similarities in these experiences support the use of generic PROMs. However, the qualitative analysis also revealed differences between patient groups that may be better captured by disease-specific PROMs.

Although for the development of completely new PROMs more traditional methods such as interviews or focus groups offer more controlled conditions, blogs can be used to assess the content validity of PROMs. This study showed that the experiences of bloggers living with breast cancer, Parkinson disease, rheumatoid arthritis, and diabetes mellitus are reflected in the domains and items of the PROMs that ICHOM recommends for these patient groups. However, potentially missing topics and potentially redundant topics could also be identified when comparing the content of PROMs with issues that were raised in blogs. Therefore, an analysis of web-based patient stories can be a useful step for researchers who are looking for PROMs that match their purposes.

## Appendix

Multimedia Appendix 1 Data collection of blogs and proms.

Multimedia Appendix 2 Codebook blogs.

Multimedia Appendix 3 Code groups aggregated in categories.

## Abbreviations

EORTC QLQ-BR45: European Organization for Research and Treatment of Cancer Quality of Life Questionnaire—Breast Cancer.
EORTC QLQ-C30: EORTC Core Quality of Life Questionnaire.
ICHOM: International Consortium for Health Outcomes Measurement
PDQ: Parkinson Disease Quality of Life Questionnaire
PROM: patient-reported outcome measure
SF-36: Short-Form 36
VBHC: value-based health care

## Data Availability

The data sets (ie, ATLAS.ti files) analyzed during this study are not publicly available because not all bloggers consented to be publicly acknowledged as authors. Publishing the original texts of their blogs would violate their anonymity as it would be very easy to retrieve their identity through search engines.
