# Supplementary material for: Using Patient Blogs on Social Media to Assess the Content Validity of Patient-Reported Outcome Measures: Qualitative Analysis of Patient-Written Blogs
Source: JMIR Form Res. 2023 Jul 28;7:e43210. doi: 10.2196/43210 (PMC10422175; doi:10.2196/43210)
Supplement: Multimedia Appendix 1 [file formative_v7i1e43210_app1.docx]

Multimedia Appendix 1. Data collection of blogs and PROMs

|  | Breast cancer patients and survivors | Rheumatoid Arthritis patients | Parkinson’s Disease patients | Diabetes Mellitus patients |
| --- | --- | --- | --- | --- |
| Source of qualitative data | Google search on “Borstkanker blog(s)” “Borstkanker reconstructie blog(s)” "Borstkanker operatie blog(s)” “Leven met borstkanker blog(s)”  Instagram search on hashtags #Borstkanker #Borstkankerblog  #Levenmetborstkanker | Google search on “blog reumatoïde artritis” “blog leven reumatoïde artritis” “reumatoïde artritis  Patiëntverhalen” “reumatoïde artritis verhaal” “reumatoïde artritis interview” “(hoe ik leef)  reumatoïde artritis” | Google search on “parkinson” AND “blog ”OR “blogs” OR “levensverhaal”OR “levensverhalen” OR “patiëntervaring” OR “patiëntervaringen” OR “verhaal” OR “verhalen” OR “ervaring” OR “ervaringen” OR “omgaan met” | Google search on “Diabetes Mellitus” “Suikerziekte blog” ‘Suikerziekte fora”  “Diabetes” “Diabetes Mellitus fora” “Diabetes Mellitus  patiënt ervaring”  “Diabetes Mellitus  blog” “Diabetes fora” “Diabetes patiënt  ervaring” Suikerziekte” “DM fora” Suikerziekte patiënt ervaring” |
| Search period | January – May 2021 | January – May 2021 | December 2019 – March 2020 | January – May 2021 |
| Inclusion and exclusion criteria | Inclusion: blogs written in Dutch by patients themselves  Exclusion: blogs written by men, by bloggers from Belgium, bloggers who write about having comorbidities, blogs that exist of less than 5 blog posts | Inclusion: blogs and patient stories of Dutch adults, diagnosed with RA and writing  about their life with RA  Exclusion: none | Inclusion: Blogs written by Dutch PD patients themselves  Exclusion: Blogs behind a paywall, blogs consisting of less than 250 words per author | Inclusion: Blogs written in Dutch by adults living with DM Type 1 or Type 2  Exclusion: Bloggers who write about having comorbidities |
| Number of bloggers | 45 bloggers, 136.000 words, 215 blog posts | 32 bloggers, 66.000 words, 75 blog posts plus patient stories based on interviews with 22 patients | 42 bloggers, 69.000 words, 114 blog posts | 40 bloggers, 55.000 words |
| PROMs recommended by ICHOM | BREAST-Q  EORTC QLQ-BR45  EORT QLQ-C30 | Numerical rating scale  Visual analogue scale  SF-36  RAND  PROMIS  Peds QL 3.0 Arthritis  Peds QL4.0 SF 15 Generic Core Scales  FACIT-F  BRAF-MDQ  HAQ-II  HAQ-DI  MD-HAQ  C-HAQ  BASFI  JAMAR  EQ-5D-5L  SF-6D  RAID  PSAID  CIHQ  WPAI | PDQ-8 | WHO-5  PHQ-9  PAID |
| PROMs included in the analysis | BREAST-Q  EORTC QLQ-BR45  EORT QLQ-C30 | HAQ  SF-36 | PDQ-8  PDQ-39 | WHO-5  PHQ-9  PAID |
| References for included PROMs | - Q-portfolio. (2021, 9 maart). BREAST-Q \| Breast Cancer. Q-Portfolio.Org. <http://qportfolio.org/breast-q/breast-cancer/> - EORTC. (2018). Specimen-BR45 English. eortc.org. <https://www.eortc.org/app/uploads/sites/2/2018/08/Specimen-BR45-English.pdf> - Bjelic-Radisic V, Cardoso F, Cameron D, et al. An international update of the EORTC questionnaire for assessing quality of life in breast cancer patients: EORTC QLQ-BR45 [published correction appears in Ann Oncol. 2020 Apr;31(4):552]. *Ann Oncol*. 2020;31(2):283-288. doi:10.1016/j.annonc.2019.10.027 - Fayers P, Bottomley A; EORTC Quality of Life Group; Quality of Life Unit. Quality of life research within the EORTC-the EORTC QLQ-C30. European Organisation for Research and Treatment of Cancer. Eur J Cancer. 2002;38 Suppl 4:S125-S133. doi:10.1016/s0959-8049(01)00448-8 - Fries JF, Spitz P, Kraines RG, Holman HR. Measurement of patient outcome in arthritis. Arthritis Rheum. 1980;23(2):137-145. doi:10.1002/art.1780230202 - Ware JE Jr. SF-36 health survey update. Spine (Phila Pa 1976). 2000;25(24):3130-3139. doi:10.1097/00007632-200012150-00008 - Peto V, Jenkinson C, Fitzpatrick R, Greenhall R. The development and validation of a short measure of functioning and well being for individuals with Parkinson's disease. Qual Life Res. 1995;4(3):241-248. doi:10.1007/BF02260863 - Topp CW, Østergaard SD, Søndergaard S, Bech P. The WHO-5 Well-Being Index: a systematic review of the literature. Psychother Psychosom. 2015;84(3):167-176. doi:10.1159/000376585 - Williams N. PHQ-9. Occup Med (Lond). 2014;64(2):139-140. doi:10.1093/occmed/kqt154 - Amsterdam UMC. (2019, January 24). PAID. Retrieved March 29, 2021, from <https://www.vumc.com/departments/diabetes-psychology/measures/paid.htm> | | | |
